# The association between anthropometric parameters, the metabolic syndrome and microalbuminuria in black Africans: the SABPA study

**DOI:** 10.5830/cvja-2010-025

**Published:** 2010-06

**Authors:** S Hoebel, JH De Ridder, L Malan

**Affiliations:** School of Biokinetics, Recreation and Sport Science, and School for Physiology, Nutrition and Consumer Sciences North-West University, Potchefstroom Campus, Potchefstroom, South Africa; School of Biokinetics, Recreation and Sport Science, and School for Physiology, Nutrition and Consumer Sciences North-West University, Potchefstroom Campus, Potchefstroom, South Africa; School of Biokinetics, Recreation and Sport Science, and School for Physiology, Nutrition and Consumer Sciences North-West University, Potchefstroom Campus, Potchefstroom, South Africa

**Keywords:** anthropometric, metabolic syndrome, microalbuminuria, black Africans

## Abstract

**Summary:**

We aimed to determine which surface anthropometric and metabolic syndrome (MS) markers could be associated with the development of microalbuminuria (MA), and assessed 200 urban Africans (25–60 years) stratified into low (≤ 0.90 and ≤ 0.85) and high (> 0.90 and > 0.85) waist-to-hip ratio (WHR) groups from the North-West province. Anthropometric and fasting MS markers, such as systolic and diastolic blood pressure (BP), and glucose, triglyceride (TG) and high-density lipoprotein (HDL) levels, as well as MA markers were measured.

Males revealed higher lifestyle risk factors (body mass index, smoking, alcohol consumption, low physical activity), anthropometric and MS markers compared to the females. The same overall trend was seen for high-WHR males but not for high-WHR females compared to their low-WHR counterparts. Both high-WHR groups revealed increased glucose values (males, 6.34 mmol/l; females, 6.13 mmol/l). Multiple linear regression analysis, independent of confounders, showed positive associations between diastolic blood pressure (DBP) (high WHR and all males), TG, waist circumference (WC) and development of MA in all males. In high-WHR females, positive associations existed only between WC and the development of MA, while neck circumference (NC) was associated with MA development in all females. To conclude, vascular BP, TG and WC were associated with risk of renal impairment in males, while in females, NC and WC circumferences were associated with this risk.

## Summary

According to Rheeder,^1^ 33 million people worldwide will have diabetes by the year 2025. Not much information is available concerning the extent of this tendency in the black African population (hereafter referred to as Africans). In terms of well being, urbanised Africans are a vulnerable group.^2^,^3^ The reason is that they have higher central obesity, blood pressure (BP) and stress levels,^4^ as well as inadequate levels of physical activity and poor dietary habits.^5^ This places them at an increased risk for developing chronic diseases such as type 2 diabetes and cardiovascular diseases,^2^,^3^,^6^-^8^ especially when they become urbanised.^9^

The increased incidence of type 2 diabetes contributes to the increased frequency of developing cardiovascular disease.^6^,^7^ Other than macrovascular complications, this increased prevalence of cardiovascular diseases in diabetic persons could be due to an increase in the metabolic syndrome (MS).^10^,^11^ Using the criteria of the World Health Organisation (WHO) the MS is represented by a cluster of risk factors which can also be present in type 2 diabetics. These factors include hypertension, high levels of blood glucose, triglycerides (TG), high-density lipoproteins (HDL), microalbuminuria and central obesity.^10^,^11^

In Caucasians, central obesity is associated with an increased waist-to-hip ratio (WHR).^11^ According to the WHO, the WHR can be seen as a risk factor to health when it exceeds 0.90 for the male and 0.85 for the female population.^11^ This measure is used as a simple method to determine body fat distribution.^12^ The MS risk can also be determined from waist circumference (WC) measurement and when it exceeds 102 cm for the males and 88 cm for the females, it becomes a risk factor.^12^ No clear cut-off points for Africans exist and it has been suggested by the International Diabetes Federation (IDF)^10^ that European anthropometric data be used as a reference when working with the African population. However, not only does data differ between ethnic groups, but there is also a difference in the type of disease prevalence between genders. African-American females have a greater tendency to present with diabetes than their male counterparts^13^-^15^ and the mortality rate is found to be higher in female diabetics than males.^13^

The information obtained from this study could be useful for screening purposes in Africans. Screening will help people to acquire the necessary understanding of the underlying factors in order to reduce or even prevent the increasing prevalence of the MS as well as other chronic diseases of lifestyle. Screening is of further use to ensure a better quality of life, and it is also cost saving in terms of disease prevention. The parameters identified during this study could be used to determine a quick and economical protocol for screening purposes in impoverished African communities. The aim of this study was, therefore, to determine which surface anthropometric and MS markers are associated with the development of microalbuminuria or renal impairment in black Africans.

## Methods

This sub-study is nested in the Sympathetic Activity and Ambulatory Blood Pressure in Africans (SABPA) study, which was a multidisciplinary target-population study conducted in 2008–2009, avoiding seasonal changes. The North-West Department of Education and the South African Democratic Teachers Union gave the necessary authorisation for this study to take place. Participants signed an informed consent form, which has been approved by the Ethics Committee of the North-West University (NWU) and the study conformed to the ethical guidelines of the World Medical Association Declaration of Helsinki.^16^

We included urban black African male (*n* = 101) and female (*n* = 99) teachers with the same socio-economic status (aged between 25 and 60 years) from one of the four Dr Kenneth Kaunda education districts in the North West province, South Africa. The exclusion criteria were: pregnancy, lactation, high temperature (> 37°C), users of α- and β-blocking agents, users of psychotropic agents, blood donors or having been vaccinated in the past three months before taking part in the study.

Collection of data for each participant continued on working days over a two-day period from February to May 2008. Participants were brought to the Metabolic Unit research facility on the NWU campus. The Metabolic Unit consists of bedrooms for each participant, bathrooms, a kitchen and a dining room as well as a living room with a television. Here the participants stayed overnight and fasted from 22:00. The following day at 06:00 the urine samples were collected followed by anthropometric measurements, which were taken in triplicate. Subsequently, blood sampling and blood pressure measurements followed.

## Equipment, measurements and analyses

Alcohol consumption and cigarette usage was determined by means of a yes/no response. The physical activity index (PAI) was determined with the help of the World Health Organisation Global Physical Activity questionnaire. A low level of activity is scored when not meeting the criteria of at least 600+ METS minutes/week.

Maximum stature was measured with a stadiometer to the nearest 0.1 cm while the participant’s head was in the Frankfort plane, the heels together and the buttocks and upper back touching the stadiometer. Mass was measured to the nearest 0.1 kg on a Krups scale with the participant wearing minimal clothes and with the weight evenly distributed. These measurements were used to calculate body mass index (BMI) by dividing weight (kg) by height (m^2^).^12^

The circumferences were measured with the participant in a standing position using a non-extensible and flexible anthropometric tape. Firstly, the neck circumference (NC) was taken immediately superior to the thyroid cartilage perpendicular to the long axis of the neck.^17^ Secondly, the WC was taken at the midpoint between the lower costal rib and the iliac crest, perpendicular to the long axis of the trunk.^17^ The hip circumference (HC) was taken at the greatest posterior protuberance of the buttocks perpendicular to the long axis of the trunk.^17^ The WHR was calculated using the WC/HC circumferences.^12^ Waist-to-height ratio (WHeiR) was calculated by dividing WC by height.^18^

Blood pressure was measured with a sphygmomanometer using the Riva-Rocci/Korotkoff method^19^ on the non-dominant arm applying the appropriate cuff size for obese and non-obese persons. Participants rested for 10 minutes in the supine position (semi-fowlers) before the first measurement was taken. Two duplicate measures were taken with a three- to five-minute resting period between each measurement and the second measurement was used for the MS criteria for BP.^10^

A fasting resting blood sample was obtained with a winged infusion set from the brachial vein branches from the right arm by a registered nurse. Glucose samples were collected in sodium fluoride tubes and all MS markers were handled according to standardised procedures and stored as serum samples at –80°C. Analysis was done using the Konelab™ 20i sequential multiple analyser computer (SMAC) (Thermo Scientific,Vantaa, Finland). Urine samples were used to determine the presence of microalbuminuria. An overnight (eight-hour) fasting urine sample of 100 ml was obtained after waking. Urine was stored at 4°C after collection and frozen at –80°C. The method involved measurement of immunoprecipitation enhanced by polyethylene glycol at 450 nm with the SMAC. HIV status was determined using antibody screening tests provided by the Department of Health in the North-West province.

## Statistical analysis

Statistical analysis was performed using the Statistica 8 program.^20^ Subjects were stratified into two groups based on their WHR (≤ 0.80 and > 0.80).^11^ The prevalence rates were computed using the two-way Pearson Chi-square analysis. For comparison between variables, analysis of covariance (ANCOVA) independent of confounders (age, BMI, PAI, smoking and alcohol consumption) was done to determine significance. Forward stepwise multiple regression analyses included MS markers (WC, glucose, TG, HDL, BP), anthropometric variables (NC) and lifestyle factors (age, BMI, smoking, alcohol, PAI) as independent variables to determine associated development of microalbuminuria. Data were regarded as statistically significant when *p* ≤ 0.05.

## Results

In Table 1, the high-WHR groups were older than their low-WHR counterparts. As reflected in the data of the total groups, males (including high-WHR males) were more inclined to exhibit higher lifestyle risk factors, such as higher prevalence rates of HIV, smoking and alcohol consumption as well as lower physical activity in comparison with their female counterparts. All females could be classified as obese with their higher BMI and WC values. Most males (including high-WHR groups) also showed a higher BMI, WC, NC and WHeiR but lower HC compared to other males and in some instances their female counterparts.

**Table 1.** Descriptive Statistics (Mean ± 95% CI): Comparing Whr Gender Groups, Independent Of Co-Variates (Age, BMI, Alcohol, Smoking, Physical Activity). Whr Values According To The WHO Definition^21^

	*Males WHR ≤ 0.90 (n = 39)*	*Males WHR > 0.90 (n = 62)*	*Total male group (n = 101)*	*Females WHR ≤ 0.85 (n = 65)*	*Females WHR > 0.85 (n = 34)*	*Total female group (n = 99)*
Age (years)	**40.79 (± 9.44)^a^**	**44.68 (± 6.7)^a^**	43.18 (± 8.05)	**43.37 (± 6.52)^b^**	**49.26 (± 8.79)^b^**	45.39 (± 7.86)
Lifestyle factors:						
AIDS (*n*) (%)	(4) 10.26	(9) 14.52	(13) 12.87	(3) 4.62	(2) 5.88	(5) 5.05
Smoking (*n*) (%)	(11) 28.21	(20) 32.26	(31) 30.69	(0) 0	(3) 8.82	(3) 3.03
Alcohol (*n*) (%)	(14) 35.9	(27) 43.55	(41) 40.59	(6) 9.23	(5) 14.71	(11) 11.11
PAI 1 (*n*) (%)	(25) 64.10	(47) 75.81	(72) 71.29	(51) 78.46	(24) 70.59	(75) 75.76
BMI (kg/m^2^)	**23.79 (± 4.46)^c^**	**29.95 (± 5.23)^c^**	**27.57 (± 5.77)^d^**	32.03 (± 7.03)	34.08 (± 7.74)	**32.73 (± 7.22)^d^**
WC (cm)	**88.74 (87.20, 90.27)^e^**	**96.59 (95.43, 97.76)^e^**	98.67 (97.14, 100.2)	**89.66 (88.13, 91.18)^f^**	**101 (98.85, 103.27)^f^**	88.36 (86.81, 89.9)
NC (cm)	**36.77 (36.07, 37.47)^g^**	**38.24 (37.71, 38.77)^g^**	**38.44 (36.88, 40)^h^**	34.45 (31.81, 37.08)	34.96 (31.15, 38.78)	**33.84 (32.26, 35.42)^h^**
HC (cm)	**102.31 (100.63, 104)^i^**	**99.90 (98.62, 101.19)^i^**	**105.44 (104.24, 106.64)^j^**	115.74 (107.84, 117.25)	110.03 (107.84, 112.23)	**109.08 (107.87, 110.3)^j^**
Waist-to-height ratio	**0.54 (0.46, 0.61)^k^**	**0.59 (0.53, 0.65)^k^**	0.6 (0.57, 0.63)	**0.57 (0.56, 0.85)^l^**	**0.63 (0.61, 0.65)^l^**	0.56 (0.53, 0.6)
Glucose (mmol/l)	**5.42 (4.72, 6.12)^m^**	**6.34 (5.81,6.88)^m^**	**6.14 (5.71, 6.57)^n^**	**4.82 (4.30, 5.35)^o^**	**6.13 (5.37,6.89)^o^**	**5.11 (4.67, 5.55)^n^**
MA (mg/l)	**9.55 (4.95, 14.15)^p^**	**13.32 (9.75, 16.89)^p^**	11.79 (9.2, 14.38)	9.92 (7.19, 12.66)	10.05 (6.06, 14.05)	10.01 (7.4, 12.64)
SBP (mmHg)	**142.74 (136.11, 149.37)^q^**	**148.78 (143.52, 154.02)^q^**	**147.56 (143.97, 151.15)^r^**	**133.88 (130.37, 137.39)^s^**	**140.89 (135.85, 145.93)^s^**	**135.11 (131.54, 138.68)^r^**
DBP (mmHg)	**84.63 (80.84, 88.42)^t^**	**86.54 (83.84, 89.54)^t^**	**86 (83.97, 88.03)^u^**	**75.66 (73.74, 77.59)^v^**	**80.05 (77.29, 82.81)^v^**	**76.96 (74.94, 78.98)^u^**
Triglycerides (mmol/l)	1.58 (1.03, 2.14)	1.96 (1.53, 2.39)	**1.77 (1.51, 2.03)^w^**	**0.88 (0.72, 1.03)^x^**	**1.33 (1.11, 1.55)^x^**	**1.08 (0.82, 1.35)^w^**
HDL (mmol/l)	1.14 (1.02, 1.27)	0.99 (0.90, 1.09)	**1.02 (0.95, 1.1)^y^**	1.2 (1.12, 1.28)	1.21 (1.09, 1.32)	**1.24 (1.16, 1.31)^y^**

CI, confidence interval; WHR, waist-to-hip ratio; BMI, body mass index; WC, waist circumference; NC, neck circumference; HC, hip circumference; MA, microalbuminuria; SBP, systolic blood pressure; DBP, diastolic blood pressure; TG, triglycerides; HDL, high-density lipoproteins. Data in bold and with superscript letters were regarded as statistically significant when *p* ≤ 0.05.

Both high-WHR gender groups (Table 1) showed increased levels of BP and impaired fasting glucose (IFG) values [males, 6.34 mmol/l (95% CI: 5.81–6.88) and females, 6.13 mmol/l (95% CI: 5.37–6.89)]. Microalbuminuria was not present (mean < 20 mg/l). High systolic (SBP) and diastolic blood pressure (DBP), as risk factors for the MS (SBP > 140 mmHg, DBP > 90 mmHg^11^), was present in both high-WHR groups. The high-WHR and total male groups presented TG values that constituted a MS risk factor (> 1.7 mmol/l).^10^ All groups presented with favourable HDL profiles (males ≥ 0.91 mmol/l and females ≥ 1.01 mmol/l).^10^

In Table 2, multiple linear regression analysis showed that the development of microalbuminuria was explained by only high vascular BP in overweight males, and by high vascular BP, TG and WC in all males. In high-WHR females, microalbuminuria was explained by increases in WC, while only NC was associated with microalbuminuria development in all females.

**Table 2.** Independent Univariate Associations Between Measures Of Microalbuminuria And Anthropometric As Well As Metabolic Syndrome Indicators

	*Microalbuminuria (mg/l)*							
	*High-WHR African males (n = 62)*		*All African males (n = 101)*		*High-WHR African females (n = 34)*		*All African females (n = 99)*	
Adjusted R^2^	0.11		0.15		0.53		0.35	
	β (SE)	*p-*values	β (SE)	*p*-values	β (SE)	*p*-values	β (SE)	*p*-values
Age	-	-	-	-	**–0.41 (0.13)**	0.00	–0.26 (0.09)	**0.01**
Smoking	-	-	-	-	-	-	-	-
Alcohol	0.13 (0.13)	0.31	0.14 (0.10)	0.18	**–0.43 (0.13)**	**0.00**	-	-
Physical activity	-	-	-	-	-	-	-	-
BMI (kg/m^2^)	-	-	-	-	**–1.18 (0.33)**	**0.00**	-	-
HC (cm)	-	-	-	-	-	-	-	-
WC (cm)	-	-	**0.40 (0.16)**	**0.02**	**0.78 (0.32)**	**0.02**	**–0.23 (0.09)**	**0.01**
NC (cm)	-	-	–0.27 (0.16)	0.10	-	-	**0.52 (0.09)**	**0.00**
SBP (mmHg)	-	-	-	-	0.21 (0.13)	0.11	0.15 (0.09)	0.11
DBP (mmHg)	**0.35 (0.13)**	**0.01**	**0.27 (0.10)**	**0.01**	-	-	-	-
TG (mmol/l)	0.16 (0.13)	0.22	**0.21 (0.11)**	**0.05**	-	-	-	-
Glucose (mmol/l)	-	-	-	-	0.21 (0.13)	0.11	-	-
HDL (mmol/l)	-	-	0.14 (0.11)	0.21	**0.37 (0.14)**	**0.012**	0.15 (0.09)	0.08

BMI, body mass index; HC, hip circumference; WC, waist circumference; NC, neck circumference; SBP, systolic blood pressure; DBP, diastolic blood pressure; TG, triglycerides; HDL, high-density lipoproteins. Data in bold were regarded as statistically significant when *p* ≤ 0.05.

## Discussion

The main aim of this study was to determine the best surface anthropometric parameters in Africans to have an association with development of the MS and microalbuminuria. In terms of lifestyle factors, it was found that the whole population had a tendency towards lower physical activity, which was associated with increased body weight and urbanisation.^3^,^8^ Inactive lifestyle and cigarette smoking are two of the risk-stratifying factors that have been set forth by the ASCM^12^ and Berger and Marais.^22^ Alcohol consumption is also known to increase BP and blood glucose levels and the risk of kidney failure, all of which are risk factors for the MS.^23^ Alcohol consumption was high in the male population, which could possibly explain its contribution to the development of these risk factors.

Urbanised Africans are more prone to being overweight or obese.^2^,^3^ Obesity was even more prevalent in the female than the male population. One reason for this difference could be attributed to the gender hormones. In males, testosterone is responsible for the high muscle mass-to-fat mass ratio,^24^ whereas oestrogen is responsible for fat distribution in females.^24^ Females also need a greater body fat percentage in order to maintain healthy living.^12^ Furthermore, the high prevalence of obesity among African females reflects their belief that overweight and obesity indicate their health status and the wealth of their spouses.^25^ The high rate of overweight and obesity found among African females in our study was consistent with the findings of Kruger *et al*.^26^ and Croft *et al*.,^27^ which stated that anthropometric measures were higher in African-American females compared to Caucasian females.

Additionally, high HC in females could be accounted for by their gynoid fat distribution.^28^ In this study, however, the females presented rather with a high WC. WC often increases with the number of births,^5^ and in older females, high WC can be a result of menopause, which changes fat distribution patterns to the android pattern.^28^ Younger females’ WC can also be attributed to water retention due to various hormonal changes during the menstrual cycle.^29^

Conway *et al*.^30^ found that WC rather than WHR should be used as measures of risk in Africans. Our study supports the finding that WC should be used, as this was positively associated with the development of microalbuminuria in males and females. Further analysis in all females (independent of obesity levels) additionally showed that NC could be used as an indicator of the development of target-organ damage or microalbuminuria. NC was related to other anthropometric parameters, which showed a relationship with metabolic disorders.^31^,^32^

The risk for development of the MS was further strengthened by the higher blood glucose levels found in both high-WHR gender groups, suggesting IFG values. Increased blood glucose levels could be due to increased insulin resistance resulting from increased accumulation of central body fat and the metabolic nature of this fat.^3^,^33^ Gender hormones have been found to affect the WC and blood glucose levels in both males and females,^34^,^35^ however, when testing for the development of microalbuminuria, glucose values did not reach significance.

In the high-WHR males, only vascular BP responses were associated with the development of microalbuminuria but in all males, the MS markers, TG and WC were also associated with increased health risk. In previous studies, we showed that the urban African male from the North-West province of South Africa has increased vascular BP and blood glucose levels.^4^ These findings have been associated with poorer physiological well being and coping with adversity in an urban environment.^4^

It has been suggested that a universal cut-off value for WHR not be used and that this value should differ between age and race groups.^36^ The IDF suggested that European data should be used when screening Africans until effective cut-off values can be developed for this population. Our study suggests that WC in males and females, as well as NC be used as a predictor of cardiometabolic risk in the African population and that Africans can be healthy and obese. African females believe that fatness reflects health,^25^ and this perceived psychological well being could contribute to actual physiological well being, possibly explaining the healthy obesity that was seen in our study. Another reason could be that some of the risk factors are heterogeneous and multifactoral;^37^ therefore anthropometry can be seen as part of the underlying cause and is not directly associated with disease prevalence.

Although neither of the groups were classified as presenting with microalbuminuria (> 20 mg/l urine), the development of renal impairment may occur as a complication of diabetes^6^,^7^,^38^ and obesity.^36^ Our study did not support this notion as we revealed only an associated trend between the development of microalbuminuria and anthropometric markers, i.e. WC in all groups and NC in females. The measured microalbuminuria levels could, however, be distorted in a random urine sample by the effects of urinary concentration and therefore a microalbuminuria:creatinine ratio is a preferable measure to lessen the variability of urine concentrations.^39^,^40^

A limitation to this study could be that the observed results were not monitored over time to determine the effect of increased or decreased anthropometric measurements on the measured physiological markers, and a follow up is recommended. In order to identify WC cut-off values for Africans, we recommend a comparison of NC, MS markers and development of microalbuminuria in WC quartiles.

## Conclusion

Vascular BP, blood triglyceride levels and WC were associated with risk of renal impairment in males, whereas in females, NC and WC circumferences were associated with this risk.
